# Non-Invasive and Minimally-Invasive Cerebral Autoregulation Assessment: A Narrative Review of Techniques and Implications for Clinical Research

**DOI:** 10.3389/fneur.2022.872731

**Published:** 2022-04-26

**Authors:** Amanjyot Singh Sainbhi, Alwyn Gomez, Logan Froese, Trevor Slack, Carleen Batson, Kevin Y. Stein, Dean M. Cordingley, Arsalan Alizadeh, Frederick A. Zeiler

**Affiliations:** ^1^Biomedical Engineering, Faculty of Engineering, University of Manitoba, Winnipeg, MB, Canada; ^2^Section of Neurosurgery, Department of Surgery, Rady Faculty of Health Sciences, University of Manitoba, Winnipeg, MB, Canada; ^3^Department of Human Anatomy and Cell Science, Rady Faculty of Health Sciences, University of Manitoba, Winnipeg, MB, Canada; ^4^Applied Health Sciences Program, Faculty of Kinesiology and Recreation Management, University of Manitoba, Winnipeg, MB, Canada; ^5^Pan Am Clinic Foundation, Winnipeg, MB, Canada; ^6^Centre on Aging, University of Manitoba, Winnipeg, MB, Canada; ^7^Division of Anaesthesia, Department of Medicine, Addenbrooke's Hospital, University of Cambridge, Cambridge, United Kingdom

**Keywords:** cerebrovascular autoregulation, computed tomography, dynamic autoregulation, magnetic resonance imaging, near-infrared spectroscopy, positron emission tomography, static autoregulation, Transcranial Doppler

## Abstract

The process of cerebral vessels regulating constant cerebral blood flow over a wide range of systemic arterial pressures is termed cerebral autoregulation (CA). Static and dynamic autoregulation are two types of CA measurement techniques, with the main difference between these measures relating to the time scale used. Static autoregulation looks at the long-term change in blood pressures, while dynamic autoregulation looks at the immediate change. Techniques that provide regularly updating measures are referred to as continuous, whereas intermittent techniques take a single at point in time. However, a technique being continuous or intermittent is not implied by if the technique measures autoregulation statically or dynamically. This narrative review outlines technical aspects of non-invasive and minimally-invasive modalities along with providing details on the non-invasive and minimally-invasive measurement techniques used for CA assessment. These non-invasive techniques include neuroimaging methods, transcranial Doppler, and near-infrared spectroscopy while the minimally-invasive techniques include positron emission tomography along with magnetic resonance imaging and radiography methods. Further, the advantages and limitations are discussed along with how these methods are used to assess CA. At the end, the clinical considerations regarding these various techniques are highlighted.

## Introduction

**Figure 1:**
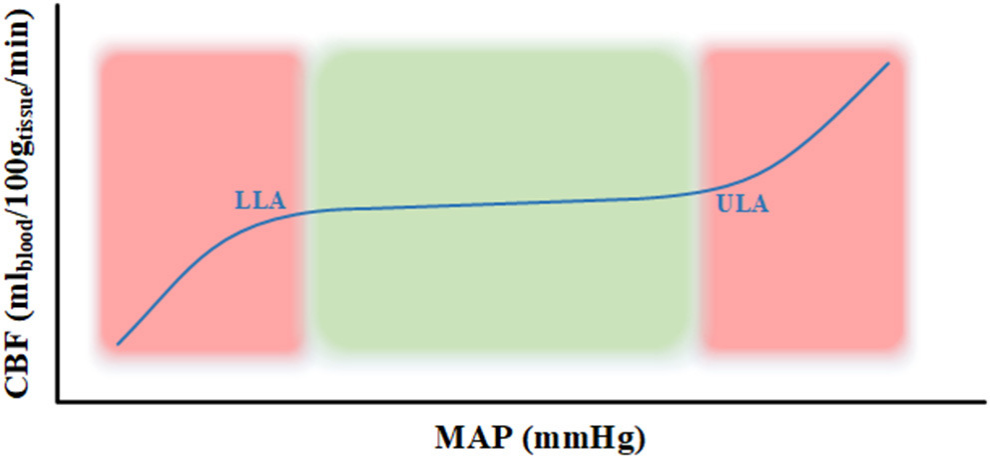
Lassen autoregulatory curve. The curve represents the Lassen autoregulatory curve (3) where the lower limit of autoregulation (LLA) and upper limit of autoregulation (ULA) are appropriately labeled. The part of the curve in the green area represents intact autoregulation and the part in the red area signifies impaired autoregulation. CBF, cerebral blood flow; g, grams; LLA, lower limit of autoregulation; MAP, mean arterial pressure; min, minute; ml, milliliters; mmHg, millimeter of mercury; ULA, upper limit of autoregulation.

The physiologic process known as cerebral autoregulation (CA) is the innate ability of the cerebral vessels to maintain a relatively constant cerebral blood flow (CBF) over a wide range of systemic arterial pressures (1, 2). Cerebrovascular reactivity (CVR) is the mechanism behind this process which occurs through the constriction and dilation of cerebral vessels to maintain a constant blood flow (1, 2). The range cerebral perfusion pressure (CPP) or mean arterial pressure (MAP) where the CA has the tendency to remain constant is between the lower and upper limits of autoregulation (LLA & ULA) on a Lassen autoregulatory curve as seen in Figure 1. When CPP/MAP goes below the LLA then the autoregulatory mechanisms are unable to adequately maintain CBF resulting in ischemia and when CPP/MAP goes above the ULA then the autoregulatory mechanisms get overwhelmed which results in hyperemia (1–3). Since CPP requires invasive ICP monitoring to derive, MAP is often used as a surrogate.

**Figure 2:**
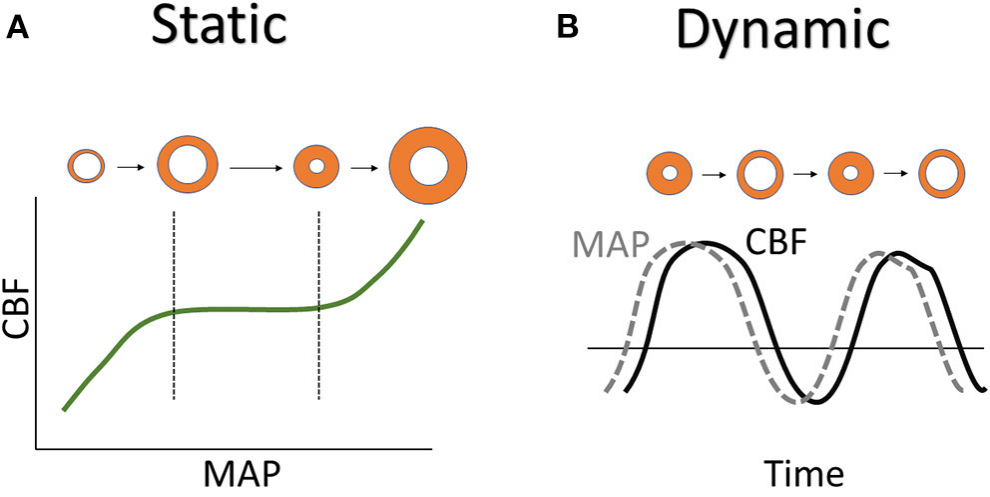
Static vs. dynamic autoregulation. **(A)** Static autoregulation is shown using Lassen autoregulatory curve with orange circles representing blood vessels state of vasodilation near the LLA and vasoconstriction near the ULA. **(B)** Dynamic autoregulation is shown by the MAP and CBF curves with orange circles representing the vasodilation and vasoconstriction of blood vessels with time. CBF, cerebral blood flow; LLA, lower limit of autoregulation; MAP, mean arterial pressure; ULA, upper limit of autoregulation.

There are two types of autoregulation measurement techniques that exists are static and dynamic autoregulation which are depicted in Figure 2. Static autoregulation evaluates CA by looking at spontaneous fluctuations in surrogate measures of pulsatile cerebral blood volume (CBV)/CBF to changes in a driving pressure when both metrics of physiologic measures have reached a steady state. Traditionally, this evaluation is performed under steady-state conditions by taking a measurement of CBV/CBF obtained at baseline CPP/MAP followed by another measurement after manipulating CPP/MAP. Dynamic autoregulation is the approach to assess CA by rapid manipulation of CPP/MAP to compare with CBF during the autoregulatory process or evaluated by using spontaneous oscillations of CPP/MAP with high temporal resolution techniques. The time scale is the main difference where static autoregulation looks at long-term change, from minutes to hours, in blood pressures while dynamic autoregulation looks at the immediate change, within seconds to minutes. Therefore, static measurements cannot address the time in which the change in CVR is achieved while dynamic measurements can, which may be relevant in certain conditions such as head injury (4, 5).

It should be noted that static vs. dynamic does not mean continuous vs. intermittent since the temporality of these techniques is not within their definitions. Continuous vs. intermittent techniques each play a role in the temporal resolution aspect of CA measurement. Continuous refers to a regularly updating measure, whereas intermittent refers to a single momentary measure or a snapshot at a point in time. Currently, there are various non-/minimally-invasive modalities that are able to assess CA in an intermittent, semi-intermittent, or continuous manner.

The focus of this narrative review is to provide an overview of the technical aspects of each non-invasive and minimally-invasive measurement techniques (i.e., cerebral non-invasive techniques that may require systemic infusions, hence minimally-invasive), highlight advantages and limitations, and look at the details behind the workings of these modalities and the methods used to assess CA. From here on, these techniques are categorized as either high spatial resolution techniques which includes neuroimaging methods or high temporal resolution techniques such as Transcranial Doppler (TCD) and near-infrared spectroscopy (NIRS). Table 1 summarizes all the techniques in the same order, as discussed in this review.

**Table 1:** Non-/Minimally-invasive cerebral autoregulation techniques summary.

**Method**	**Technique**	**Overview**	**Advantages**	**Disadvantages**	**CA assessment**
**Dynamic techniques**					
Biomedical Optics	Spatially-resolved NIRS (6–9)	Uses multiple light detectors to measure the attenuation of NIR light at different wavelengths on the illuminated tissue	• Extracranial circulation can be eliminated with short path light detectors	• Gives relative concentrations of chromophores instead of absolute concentration, scattering parameters are assumed in the calculation	Continuous
	Frequency-resolved NIRS (10)	Uses an intensity-modulated light source to make a measurement of the detected light intensity, then determines the phase shift and modulation depth with respect to the input light	• Provides absolute quantification of hemodynamics	• Less information about the tissue is provided if only one frequency is used	Continuous
	Time-resolved NIRS (11–13)	Uses a picosecond laser source to input ultrashort pulses of light into the tissue and detects the intensity of emergent light from the tissue as the TPSF with picosecond resolution	• Contains information about changes in light absorption at different depths	• Dynamic range of a system with a streak camera is limited, along with it being expensive and large in size • System with time-correlated photon counting system detector is limited by its low speed	Continuous
	fNIRS (14)	Uses separate light sources and detectors with a varying source-detector distance to assess regional tissue oxygenation	• Ability to incorporate short separation channels to remove scalp noise • Portable and cost-effective	• Gives relative concentrations of chromophores instead of absolute concentrations	Continuous
	DCS (15, 16)	Uses intensity temporal autocorrelation function based on detected photon arrival times to quantify the blood flow and provide direct measure of CBF	• Provides a direct measure of CBF • Ability to be combined with frequency- and time-resolved NIRS	• Light collection is sensitive to the presence of hair	Continuous
Ultrasound	TCD (4, 17–21)	Leverages the principle of Doppler effect to determine CBFV by looking at the Doppler shift in frequency of ultrasonic waves at a known frequency emitted by ultrasound probes, through one of the naturally occurring acoustic windows and reflected off by moving RBCs in the vessel of interest	• Relatively inexpensive • A portable machine which can be used at the bedside • Has various autoregulation indexes	• Typically limited to <60 min of recording, but special care is required to perform longer recordings	Semi-intermittent
MRI	DWI (22)	Brownian motion is used to generate contrast using specific MRI sequences along with software in images	• Does not require administration of any contrast agent	• Sensitive to involuntary motion • Expensive	Intermittent
	PWI DSC	Uses various MRI sequences with intravenous administration of a bolus of gadolinium-based contrast agent that is monitored through the brain tissue using T2- or T2*-weighted images. Concentration-time curves can be obtained from signal information to derive quantitative parameters such as CBV, CBF, and MTT	• Most widely used method to measure brain perfusion • Higher SNR	• Minimally-invasive techniques due to administration of a bolus of gadolinium-based contrast agent given intravenously • Expensive	Intermittent
	PWI ASL	Uses magnetically labeled blood, with a combination of radio-frequency pulse and a field gradient, as a freely diffusible tracer for CBF measurements	• Non-invasive technique since it uses magnetically labeled blood as the tracer	• Sensitive to potential motion artifacts • Overall, SNR is limited • Expensive	Intermittent
	fMRI (23)	BOLD fMRI describes time-varying changes in brain metabolism via changes in HHb concentration since HHb is able to slightly distort the magnetic field in its vicinity as compared to HbO	• BOLD is used as the non-invasive contrast that results from change in magnetic field surrounding RBCs based on hemoglobin's oxygen state	• Sensitive to potential motion artifacts • Expensive	Intermittent
**Static techniques**					
Nuclear Medicine	PET (17, 24–29)	Uses small amounts of radioactive material injected intravenously to construct series of projections representing distribution of regional radioactivity view from different angles with the help of detector pairs and data processing software on a computer	• Scanners with multiple rings of detectors allow simultaneous reconstruction of several slices at different levels of the brain	• Minimally-invasive since it requires radiotracers injected intravenously	Intermittent
Radiography	CTP (17, 30–33)	Uses X-rays, intravenous iodinated contrast agent and a computer to acquire time-concentration curves of ROI. Relies on the central volume principle and the manipulation of these curves to calculate standard CTP measures	• Less time consuming • Does not require any special equipment other than a post-processing software	• Minimally-invasive since a bolus dose of an iodinated contrast agent needs to be administered	Intermittent
	Xe-CT (17, 34–39)	Uses inhalation of a gas mixture containing xenon with X-rays and a computer to assess change in image attenuation during wash-in and wash-out phases of xenon	• Greater brain coverage	• Minimally-invasive since inhalation of a gas mixture is required • Motion can create artifacts	Intermittent

*ASL, arterial spin labeling; BOLD, blood oxygen level dependent; CA, cerebral autoregulation; CBF, cerebral blood flow; CBFV, cerebral blood flow velocity; CBV, cerebral blood volume; CTP, computed tomographic perfusion; DCS, diffuse correlation spectroscopy; DSC, dynamic susceptibility contrast; DWI, diffusion weighted imaging; fMRI, functional magnetic resonance imaging; fNIRS, functional near-infrared spectroscopy; HbO, oxyhemoglobin; HHb, deoxyhemoglobin; MRI, magnetic resonance imaging; MTT, mean transit time; Mx_a, mean flow index with arterial blood pressure; NIR, near-infrared; NIRS, near-infrared spectroscopy; PET, positron emission tomography; PWI, perfusion weighted imaging; RBC, red blood cell; ROI, region of interest; SNR, signal to noise ratio; TCD, transcranial doppler; TPSF, temporal point spread function; Xe-CT, Xenon-computed tomography*.

## Radiography

Imaging techniques using X-rays, or similar ionizing or non-ionizing radiation to non-invasively view inside various regions of interest (ROI) is known as radiography. Computed tomography (CT) is a modality in medical radiography that uses ionizing radiation in combination with a computer to create images of the ROI. Two techniques that evaluate CBF using radiography are computed tomographic perfusion (CTP) (40, 41) and Xenon-CT (Xe-CT) (41, 42).

### Computed Tomographic Perfusion

CTP relies on the central volume principle that relates CBF, CBV, and mean transit time (MTT), as seen in Equation 1. It is conducted by first administering a bolus dose of iodinated contrast agent followed by assessing the transient increase in attenuation proportional to the amount of given contrast agent since there is a linear relationship between contrast agent concentration and attenuation (40). The contrast agent time-concentration curves are generated for arterial, venous, and parenchymal ROI. By manipulating these curves, we can calculate the CTP's standard measures which are CBF, CBV, MTT, and time to peak (TTP) (17, 40, 41, 43). CTP can be used for intermittent static autoregulatory assessment by acquiring two scans. The first scan is obtained as a baseline and then the second scan is obtained after deliberate manipulation of systemic blood pressure that targets a specific CPP/MAP, which is typically 20 mmHg above baseline (17, 30–33). An increase in CBF with an increase in CPP indicates disrupted CA whereas intact autoregulation is depicted by CBF independent of CPP values (17, 33).

Equation 1:


(1) $$\begin{array}{l} MTT=\frac{CBV}{CBF} \end{array}$$

### Xenon-CT

Compared to CTP, where CBF is estimated by changes after intravenous iodinated contrast, Xe-CT is based on CT scanning during inhalation of a gas mixture containing ~30% xenon for a total duration ranging from 3 to 6 min (17, 41, 42). By being able to assess the change in image attenuation during wash-in and wash-out phases of xenon with CT and the estimated arterial xenon levels, assumed to be equal to end-tidal levels from xenon detector, the CBF can be calculated using Equation 2 and Equation 3. These two equations are known as the modified Kety-Schmidt equations where *C*_*b*_(*T*) is the time-dependent brain xenon concentration, λ is the brain-blood partition coefficient, *K* is the brain uptake flow rate constant, and *C*_*a*_(*t*) is the time-dependent arterial xenon concentration (41–43). With Xe-CT, intermittent static autoregulation can be assessed in a similar manner as done with CTP where a baseline Xe-CT is obtained then Xe-CT is repeated after a target CPP/MAP manipulation is reached (34–39). Similarly, “intact” autoregulation is defined when there is little to no change in CBF during manipulation of CPP/MAP otherwise the autoregulation is considered “impaired.” Since studies have defined varying thresholds, it is not clear which thresholds definitively are associated with autoregulatory dysfunction and which are not (17).

Equation 2:


(2) $$\begin{array}{l} \begin{array}{c} C_{b}(T)=\;\text{λ}K\int_{t=0}^{T}C_{a}(t)e^{-K(T-t)}dt \end{array} \end{array}$$

Equation 3:


(3) $$\begin{array}{l} CBF=\text{ λ}K \end{array}$$

CTP studies are less time consuming, and do not require special equipment other than a dedicated post-processing software as compared to Xe-CT studies (41). While Xe-CT has a greater brain coverage since the iodinated contrast material used in CTP has much quicker kinetics (41), Xe-CT has limitation of patient motion which can produce artifacts in the images (43). Both CTP and Xe-CT have been shown to have a good correlation between their results (41). Also, both of them are static measures of autoregulation that are measured intermittently.

## Magnetic Resonance Imaging

Magnetic Resonance Imaging (MRI) is a non-invasive medical device that uses powerful magnets, usually 0.5T to 3T, to produce detailed three-dimensional images of the body by using its natural magnetic properties. Hydrogen nuclei are used for imaging purposes due to their abundance in water and fat (44).

To obtain an MRI image of the desired part of the body, it is placed inside an MRI scanner that creates a strong magnetic field using powerful magnets, causing proton's axes in the exposed tissue to align uniformly along the direction of the magnetic field, creating a magnetic vector. With the addition of energy in the form of radio waves, the magnetic vector gets temporarily deflected. The MRI image is created on a greyscale image by plotting intensity of the energy released in the form of radio wave signals by the protons returning to baseline when the radio frequency source is turned off. There are two ways, T1 and T2, that the relaxation of protons is measured. T1 relaxation is the time the magnetic vector takes to return to its resting state andT2 relaxation is measured by the time needed for an axial spin to return to its resting state. The magnetic field can be altered electronically by large gradient electric coils along with varying the applied radio frequencies to isolate different slices of the body (44). CBF can be assessed using MRI techniques such as Diffusion-Weighted Imaging (22, 45), Perfusion-Weighted Imaging (17, 43), and functional MRI (fMRI) (17). These techniques are dynamic measures of autoregulation which are measured intermittently.

### Diffusion-Weighted Imaging

A method that uses differences in Brownian motion to generate contrast using specific MRI sequences along with software in images from resulting data is Diffusion-Weighted Weighted Imaging (DWI). Brownian motion is the random movement of water molecules in a medium. In the presence of obstacles, such as cell membranes, the water molecules get confined which restricts the normal free diffusion. A large percentage of the human body is composed of water and in this complex environment, water molecules experience free diffusion and restricted diffusion in extracellular and intracellular environments, respectively (46, 47). An initial validation study has shown that following thigh cuff deflation, the estimated changes in cerebral perfusion using the MRI DWI technique is at least as reliable as the classical TCD technique, which has been viewed as a standard method to assess dynamic CA (22). Also, MRI have been shown to give more consistent measures than TCD (22).

As outlined by Saeed et al. (22) dynamic CA can be assessed using the MRI DWI technique and the imaging series begins when thigh cuffs get inflated 20 mmHg above the peak systolic ABP. Rapid serial acquisition occurs using a gradient-echo echo-planar imaging sequence. After 3 min, the cuffs are rapidly deflated to introduce a transient blood pressure (BP) drop and the procedure would be completed at 4 min. By acquiring 240 multi-slice image sets over 4 min gives the minimum temporal resolution of 1 s. Then visual inspection of movement can occur by playing them in a movie loop after having the dynamic images composed into 4-dimensional sets (3 spatial dimensions and 240 time points) by using a software package designed specifically for processing MRI. The analysis occurs on the four central slices corresponding to the main anatomic areas perfused by the middle cerebral artery (MCA). On each of the four slices, the brain ROI is divided into left and right halves to produce a total of eight regions of interest. Then the change in signal intensity is extracted for each of the eight ROIs over the 240-s time series, and an average of the signal intensity for the four slices is calculated to get the mean intensity time course for the left and right sides of the brain. With the described MRI data acquisition and analysis, it has been shown that following thigh cuff release, MRI intensity changes however, to what extent this is a reliable index of dynamic CA remains to be shown (22).

Compared to the conventional DWI image, an Apparent Diffusion Coefficient (ADC) image, also known as ADC map, allows the magnitude of water diffusion to be quantified (45, 46). ADC scalars, which represents the lowest mean of three contiguous axial sections between the left and right cerebral hemispheres, have been shown to significantly correlate with blood pressure autoregulation in the posterior centrum semiovale, posterior limb of the internal capsule and globus pallidus regions of each cerebral hemisphere in neonates cooled for perinatal hypoxic-ischemic injury (45).

### Perfusion-Weighted Imaging

For studying cerebral hemodynamics, Perfusion-Weighted Imaging (PWI) is an established MRI method that gives insights into the perfusion of tissues by blood. The PWI techniques use various MRI sequences with and without the administration of contrast agents to acquire signals that are used to generate perfusion maps with parameters such as CBF, CBV, and MTT with post-processing. The main techniques that come under the PWI method are Dynamic Susceptibility Contrast (DSC), and Arterial Spin Labeling (ASL) (47). With PWI, dynamic autoregulation could be assessed in a similar manner as described in the DWI section where thigh cuffs are inflated to a pressure for couple minutes before the cuffs are rapidly deflated in the last minute, introducing a transient BP drop. The acquisition of slices would be done intermittently for the dynamic autoregulation measure but the assessment of dynamic autoregulation with PWI MRI techniques has not been done.

#### Dynamic Susceptibility Contrast

The MRI DSC techniques are the most widely used method to measure brain perfusion but are minimally-invasive. They require the administration of a bolus of gadolinium-based contrast agent intravenously, which is monitored through the brain tissue using T2- or T2^*^-weighted images. From the signal information, concentration-time curves can be obtained to be able to derive important quantitative parameters such as CBV, CBF, and MTT. In general, DSC perfusion achieves a higher signal-to-noise ratio (SNR), allowing images to be at a higher temporal and spatial resolution (47, 48).

#### Arterial Spin Labeling

The MRI ASL technique uses magnetically labeled blood as an endogenous diffusible tracer for CBF measurements. It is a non-invasive technique since it does not use contrast agents, thus labeling is achieved by inverting the blood magnetization with a combination of radio-frequency pulse and a field gradient in the direction of blood flow (47, 48). The two main types of ASL techniques are continuous ASL and pulsed ASL. In continuous ASL technique, arterial blood water underneath the imaging slab is continuously labeled with prolonged radiofrequency pulse until a steady-state tissue magnetization is reached, whereas pulsed ASL technique is less demanding in the sense that at a single point in time, a short radiofrequency pulse labels arterial blood and it is allowed to distribute to the tissue of interest before imaging is performed. Theoretically, CBV and MTT can be obtained using ASL but these methods are not widely used. The overall SNR of ASL is limited due to sensitivity to potential motion artifacts, but it could be improved with using high-quality and high field strength scanners. Overall, the acquisition times are longer due to the limited SNR and in part, explain the lower utilization of ASL as compared to DSC (48).

### Functional MRI

Blood oxygen level dependent (BOLD) functional MRI (fMRI) describes time-varying changes in brain metabolism via changes in deoxyhemoglobin (HHb) concentration. Since fMRI is based on MRI, it can incorporate various types of contrast such as T1 weighting, T2 weighting, etc. without the injection of any radioisotopes. BOLD is known as the contrast which results from a change in magnetic field surrounding the red blood cells (RBCs) based on the oxygen state of the hemoglobin. Oxygenated hemoglobin or oxyhemoglobin (HbO) is almost resistant to magnetism (diamagnetic) hence it is indistinguishable from brain tissue while HHb is more magnetic (paramagnetic) and is able to slightly distort the magnetic field in its vicinity. Hence, the variations in regional tissue oxygenation due to changes in oxygen uptake and altered blood supply caused by local brain activity can be mapped by T2^*^ weighted MRI (47, 49). By utilizing gradient refocused echo (GRE), BOLD fMRI is able to increase T2^*^ contrast (49). It has been proposed that resting-state fMRI, which measures spontaneous low-frequency fluctuations in BOLD signal, looks promising for assessing dynamic CA with high spatial resolution since it has not extended into this domain yet (23).

## Positron Emission Tomography

Positron emission tomography (PET) is a type of nuclear medicine imaging, also known as PET imaging or PET scan, which uses small amounts of radioactive material called radiotracers to help diagnose and assess medical conditions. PET imaging requires three components: a positron-emitting isotope (radiotracer), a tomographic imaging system to detect the location along with measure quantity of radiation, and lastly, a mathematical model which is often needed to relate the physiologic process under study to the detected radiation (50, 51). The PET imaging technique is minimally-invasive due to the intravenously injected radiotracers, and since PET utilizes ionizing radiation, it is not entirely benign.

Radiotracers are radioactive molecules, or radionuclides, that do not affect the physiologic process being studied by administering them in small quantities. PET radiotracers can be separated into two broad categories: normal biologic molecules (^11^C, ^13^N, ^15^O) or non-biologic elements that can be attached to organic molecules as radiolabels (^18^F, ^68^Ga, ^75^Br). The half-life of these tracers range from a couple of minutes to a couple of hours, and they decay by positron emission (50, 51).

The second component of PET imaging is the imaging system which consists of a large number of detector pairs to localize and quantify the brain's physiologic processes by using the phenomenon of annihilation radiation. Radionuclides undergo positron emission decay where it emits a positron, a positively charged electron. This emitted positron may travel for a short distance in the tissue, usually a few millimeters depending on the isotope, losing energy before it can encounter an electron. The encounter between the positron and an electron results in annihilation of both and results in the generation of a pair of gamma photons, of equal energy, moving in approximately the opposite direction. These two photons are detected simultaneously by a pair of detectors positioned on either side from the source of annihilation, which allows localization of the radiation's point of source (50).

Using a computer, the data from the detector pairs is used to construct a series of projections that represents the distribution of regional radioactivity viewed from different angles. By combining the projections, a two-dimensional image is produced while a three-dimensional image can be produced with scanners containing multiple rings of detectors, allowing them to generate several reconstructed slices of imaged volume simultaneously where each depicts a different level of the brain (50).

Some of the cerebral physiological factors that can be measured by PET are CBF, CBV, and MTT. Most commonly, ^15^O-labeled water (${\text{H}}_{2}^{15}$O) is used as the intravenous tracer agent to measure CBF by obtaining the emission scans and calculating CBF maps using kinetic models that treat water as a freely diffusible tracer. The measurement of CBV is done in a similar fashion using ^15^O-labeled carbon monoxide (C^15^O) as the tracer agent (17, 50). MTT is not directly measured by PET but by the central volume theorem, MTT can be calculated as the ratio of CBV to CBF as seen in Equation 1 or its inverse, which hypothetically is the particle's mean time to pass through the cerebral circulation (50). With the CBF and CBV measurements, PET-based techniques can assess autoregulatory capacity by first obtaining the CBF maps at baseline physiology and after a change in CPP from baseline. With little or no change in CBF/CBV during CPP manipulations, autoregulation is defined as “intact” while an increase in CBF/CBV during CPP elevation is defined as “impaired” autoregulation (17, 24–29). This is a measure of static autoregulation conducted intermittently. PET can be combined with CT to produce special views, and an emerging technology of interest is the combination of PET and MRI techniques.

## Transcranial Doppler

Transcranial Doppler (TCD) is a type of ultrasonography that measures CBF velocity (CBFV) in real-time by using the principle of the Doppler effect (5). First observed by Christian Andreas Doppler in the mid-1800's, the Doppler effect is the phenomenon where a soundwave, at a certain frequency, strikes an object in motion and is reflected with a different frequency (52). The echo will be of a higher frequency if the object is moving toward the source of the soundwaves, and the echo will be of a lower frequency if the object is moving away from the source. The Doppler shift is known as the difference of the emitted frequency vs. the reflected soundwave and it is directly proportional to the relative speed of the moving object to the source (18).

The principle of the Doppler effect has been leveraged to determine CBFV by having the TCD ultrasound probes emit an ultrasonic wave at a known frequency through the skull which gets reflected off RBCs in the vessel of interest, producing an echo at a different frequency (5, 18). This Doppler shift in frequency allows for the calculation of the CBFV but not proper CBF although both CBF and CBFV have a linear relationship over physiologic ranges as long as the vessel diameter is constant (18). Due to the cranial cavity being fully encased by the cranial bones in an adult, the ultrasonic signal gets heavily attenuated by the bone tissue resulting in only 6% of the ultrasonic signal reaching the brain (52). To properly insonate the cerebral arteries, there exists naturally occurring thinner areas of bone known as acoustic windows that allow for the best transmission of the TCD signal. The four naturally occurring acoustic windows are transtemporal, transorbital, transforaminal or suboccipital, and submandibular windows. The transtemporal window can be used to study the anterior cerebral arteries, middle cerebral arteries, and posterior cerebral arteries. The transorbital window can be used to examine the ophthalmic artery and cavernous portion of the intracranial carotid artery (ICA). The transforaminal window, also known as the suboccipital window, allows the examination of the basilar artery and vertebral arteries. Finally, the suboccipital window can be used to study the distal ICA (5, 52).

We can get a better understanding of how the ultrasound technology, used in TCD, is based on the Doppler effect by looking at Equation 4, which gives the calculation of the reflector velocity, *v*, of the moving target. A TCD probe functions by emitting an ultrasonic wave at a known frequency, *f*_*o*_, moving through the tissue at a speed, *c*, and then receiving the echo. The echo is produced at a different frequency, *f*_*e*_, resulting from the wave reflected off the moving RBCs in the vessel of interest. The Doppler shift, *f*_*d*_, is determined by *f*_*d*_ = *f*_*e*_ − *f*_*o*_. The velocity is corrected by the Doppler angle or the angle of insonance, θ, which should be <30° to have the maximum degree of error <15% (5, 18, 52). The parameters that can be derived from TCD include flow velocity (FV) and pulsatile index where the FV can be distinctly described as peak systolic flow velocity (FVs), end-diastolic velocity (FVd) and mean velocity (FVm) (18). CA can be assessed using TCD but due to the practical limit of TCD monitoring of less than an hour restricts the TCD based methods to intermittent and semi-intermittent. The TCD-based intermittent methods are such as rate of regulation (RoR), autoregulatory index (ARI), transient hyperemic response testing (THRT) and orthostatic hypotension test (OHT) while the semi-intermittent techniques are mean flow index, systolic flow index and diastolic flow index (17, 18).

Equation 4:


(4) $$\begin{array}{l} v=\frac{c\;*\;\left(f_{e}-f_{o}\right)}{2\;*\;f_{o}\;*\;\cos\theta }=\frac{c\;*\;f_{d}}{2\;*\;f_{o}\;*\;\cos\theta } \end{array}$$

RoR can be calculated from the data obtained by the thigh cuff deflation technique (TCDT) that was first described in 1989. By inflating bilateral thigh cuffs for 2 min and rapidly deflating them, it induces a step decrease in ABP. The time interval is measured until the FV returns to baseline and RoR is calculated by Equation 5, where normal RoR is characterized as 0.2/s (53). The ARI method is built on RoR using a second-order differential model of CA constructed with three physical properties which are the time constant, the damping factor and the autoregulatory gain. The strength of autoregulation is graded on an ordinal scale from zero to nine, where zero corresponds to the total loss of autoregulation and nine corresponds to the hyperactive regulatory response. During the change in MAP, the TCD based FV changes are normalized and compared to model-generated responses corresponding to the 10 grades and the best fit is deemed the ARI. ARI of 4-7 is defined as normal CA, ≤3 corresponds to loss of autoregulation and ≥8 corresponds to hyperactive regulatory response (4). THRT is another method of assessing CA by the compression of the carotid artery in the neck while the ipsilateral MCA is insonated. The carotid artery is released after 3 s of stable reduction in FV, where an adequate reduction is 30–50% in FV and the changes in MCA FV are measured with TCD. CA is suggested to be intact if the overshoot of FV is at least 10% of baseline FVs while overshoot of <10% is suggested to represent impaired CA (19). Another way to reduce CPP is by the rapid change in head position in the Orthostatic hypotension test (OHT). With rapid elevation of a patient's head position under <3 s, a sudden drop in CBFV can be evaluated by the TCD. If the reduction of CBFV as compared to baseline is between 10 and 15% then it is considered normal CA, while a drop in CBFV of >20% indicates disrupted autoregulation (20, 21). Most significant limitation of the intermittent methods is that an eternal perturbation is required to the subject's physiologic parameters to assess CA (18).

Equation 5:


(5) $$\begin{array}{l} RoR=\frac{\text{Δ}\left(ABP/CBF\right)}{\text{Δ}t\;*\;\Delta MAP} \end{array}$$

Looking at assessment of more continuous CA using slow wave changes in MAP, these methods work with having a correlation coefficient between some surrogate of CBF and some measure of driving pressure, regularly updated, over an advancing set time window. These correlation coefficients, or indices, have three different variations MAP as a surrogate for driving pressure. These variations are mean flow index with arterial blood pressure (Mx_a), systolic flow index with arterial blood pressure (Sx_a), and diastolic flow index with arterial blood pressure (Dx_a) where FVm, FVs, and FVd are respectively used as their CBF surrogates. All these indices have driving pressure and FV processed with a 10-s moving average filter, and the correlation coefficient is calculated using a 5-min window, updated every 10 s or every minute depending on the recording time length. The values of the index are normalized to values between −1 and +1 where more positive values suggest more dysfunctional CA. These three indices are different than their counterparts, Mx, Sx, and Dx, that use CPP as a surrogate for driving pressure and require the use of invasive intracranial monitoring. Preliminary studies seem to indicate that Mx_a and Sx_a can be used to estimate ICP based indices of CVR such as pressure reactivity index (PRx), although Dx_a showed no significant predictive value (18). Although, early TCD-based works assessed static autoregulation, currently it is commonly used to assess dynamic autoregulation conducted semi-intermittently, since longer recording periods require very special care.

## Near-Infrared Spectroscopy

A non-invasive modality based on near-infrared (NIR) light that is gaining traction to assess CA is the near-infrared spectroscopy (NIRS). NIR light is a region of the infrared spectrum where the wavelengths of light ranges from 650 to 950 nm and this region has the lowest amount of light absorbed by water molecules and hemoglobin. Below the range, visible light is strongly absorbed by hemoglobin and above the range, the absorption by water molecules increases significantly (54). To observe blood and tissue oxygenation using NIR light, Modified Beer-Lambert Law (MBLL) is used which describes absorption of light in a multiple scattering medium, such as tissue, where light takes a longer path (11). MBLL is given by Equation 6 where ε is the molar extinction coefficient that gives the absorption of light in the medium, C is the concentration of the medium, d is the optode spacing, DPF is the differential pathlength factor quantifying the additional pathlength attributed to scattering, and G describes the tissue's scattering coefficient with the optode geometry (11, 55).

Equation 6:


(6) $$\begin{array}{l} A=\varepsilon *C*d*DPF+G \end{array}$$

NIRS devices contain optical probes that are placed on the scalp to obtain measurements of CBF. The probes contain a combination of transmitter and receivers where the transmitter is used to transmit NIR light into the scalp and the receivers detect the NIR light after it has gone through scattering and absorption. Three main naturally occurring chromophores, or absorbers, of interest are HbO, HHb, and Cytochrome C Oxidase (CCO). The measurements for each chromophore are performed by calculating the change in attenuation with different wavelengths using MBLL.

There are different types of NIRS available that make use of different technologies to provide continuous bedside cerebral physiologic measurements. All of them measure dynamic autoregulation by rapidly manipulating MAP by techniques such as thigh cuff deflation of sit-to-stand and the NIRS data is recorded continuously. We take a look at continuous wave, spatially-resolved, frequency-resolved, time-resolved, diffuse correlation spectroscopy, and functional near-infrared spectroscopy.

### Spatially-Resolved NIRS

The spatially-resolved NIRS contains multiple light detectors in a probe that are at different distances from the constant intensity NIR light transmitter. The use of multiple light detectors is able to eliminate the extracranial circulation from the scalp, recorded by short path light detector, from the longer path light detectors which penetrate deeper into the brain tissue. It is able to derive the relative concentrations of HbO and HHb along with estimating mean tissue hemoglobin saturation by measuring the attenuation of NIR light at different wavelengths using the multiple light detectors on the illuminated tissue (56).

Some spatially-resolved NIRS, like NIRO devices, can derive tissue oxygenation index (TOI) and total hemoglobin index (THI) along with relative concentrations of HbO, HHb, and CCO (6). The ratio of HbO to the total tissue hemoglobin (HbT) gives TOI (7) while THI is a surrogate for HbT of the measured tissue and is expressed as arbitrary units since the exact total volume of tissue is not known (6). Like TOI, regional cerebral oxygen saturation (rSO_2_) is another measure for cerebral tissue oxygenation reported by INVOS devices. However, it is still unclear if THI and rSO_2_ can be used interchangeably since the proprietary methodologies and assumptions in the calculation of cerebral tissue oxygenation varies between manufacturers (8).

PRx has been validated to accurately detect LLA in animal models (57) and is considered a “gold standard” for continuous bedside assessment of CVR (29) but it requires invasive ICP measure. NIRS based indices of CVR have been developed that substitute NIRS based metrics in place of ICP. These indices are total hemoglobin index (THx), tissue oxygen index (TOx), and cerebral oximetry index (COx) that use the NIRS based metrics THI, TOI, and rSO_2_, respectively. These NIRS based indices have shown to correlate well with PRx and TCD based indices as found by Smielewski and colleagues in a mixed cohort of 150 patients where 40 patients had severe TBI, 27 patients had subarachnoid hemorrhage (SAH), 60 patients were undergoing cardiopulmonary bypass, and 23 patients had sepsis (9).

### Frequency-Resolved NIRS

The frequency-resolved NIRS contains either LED or laser diode as the light source which is intensity-modulated at radio frequencies to provide information on the scattering properties of the medium. These measurements from the detected light intensity of the output light along with the phase shift and modulation depth, with respect to input light, can provide absolute quantification of hemodynamics (58). There exists a linear relationship between frequency and phase shift for the light transmitted through tissues up to 200 MHz but this relationship is not present at higher frequencies (59). Regional cerebral tissue oxygenation (cStO_2_) can be derived from frequency-resolved NIRS such as INVOS 5100c and FORE-SIGHT. It is challenging to define normal ranges of cStO_2_ due to the difference in the absolute values between commercial devices (10). These frequency-resolved systems are also referred to as frequency domain, intensity-modulated or phase-resolved systems.

### Time-Resolved NIRS

The time-resolved NIRS contains information on the different depths of light absorption since photons from deep tissue paths will arrive later than photons from superficial paths (60). These systems are equipped with a picosecond laser source that generates ultrashort pulses of light into the tissue for detecting temporal point spread function (TPSF) with picosecond resolution, which is the measurement of the intensity of the emergent light from the tissue (11, 12). The ultrafast detectors used in these systems are streak camera giving high temporal resolution or time-correlated photon counting system that provides a wide dynamic range while using cheaper components. Along with detecting TPSF, time-resolved NIRS such as TRS-10 can provide measurements of oxygen saturation (SO_2_) (13). These time-resolved systems are also referred to as time-domain or time-of-flight systems.

### Functional Near-Infrared Spectroscopy

Functional near-infrared spectroscopy (fNIRS) is another non-invasive bedside tool to continuously assess regional tissue oxygenation. Unlike fMRI, fNIRS measures both hemoglobin, HbO and HHb, separately along with being portable and cost-effective. Typically, fNIRS systems use separate light sources and detectors with a source-detector distance ranging from 1.5 to 3.5 cm. In recent years, short separation channels source-detector separation of <1 cm has been incorporated to remove the scalp noise in studies. Also, there has been development of multichannel fNIRS, which increases the spatial resolution of this technology (14).

### Diffuse Correlation Spectroscopy

Diffuse Correlation Spectroscopy (DCS) is an emerging optical technique that can provide a direct measure of CBF by quantifying the blood flow using temporal fluctuations of the reflected NIR light (61). These fluctuations are caused by moving RBCs scattering the light that can be quantified by intensity temporal autocorrelation function based on the detected photon arrival times (61, 62). Information about the metabolic rate of oxygen can be determined by pairing DCS with any NIRS system. It has been demonstrated that by combining DCS with frequency-resolved NIRS, we can quantify cerebral tissue oxygen metabolic rate (CMRO_2_) from the combination of oximetry and flow measures (15). DCS can also be combined with time-resolved NIRS to measure absolute CMRO_2_ and it has been verified against cerebral venous blood sample derived values of CMRO_2_ (16). Current limitation of DCS is that the light collection is sensitive to the presence of hair but it can be tackled by the use of many fibers in a single spot (61).

## Clinical Considerations

There are numerous considerations that the bedside clinician needs to consider when determining the appropriate modality for monitoring CA. The first is patient safety, as each modality has varying safety profiles. CT and PET-based modalities come with the small, but not insignificant, risk of ionizing radiation, which may lead to DNA damage and ultimately malignancy (63, 64). CTP additionally carries an additional risk as it requires iodinated contrast agents which are nephrotoxic and are a potential anaphylactogenic (65). While MRI-based methods are free from ionizing radiation, some modalities do require the administration of nephrotoxic contrast agents (66). Additionally, the requirement of large magnetic fields in MR imaging makes it unsafe for those with certain implanted medical devices such as pacemakers (67). In the clinical setting, given the lack of availability of portable CT and MRI scanners, these modalities necessitate the transportation of the patient to the machine. This may be tolerated in most but is not without risk in critically ill patients (68).

A number of modalities require the measurement of ICP or its derivative CPP. These methods require the invasive placement of a probe into the cranium, which carries the risk of brain injury through hemorrhage or infection (69). Typically, however, these indices are implemented in situations where ICP monitoring is already indicated, such as moderate and severe traumatic brain injury (TBI), and thus do not present additional risk (70).

Beyond the various surrogates for CBF, modalities that require manipulation of ABP or cerebral hemodynamics might also not be well tolerated by various groups of medically ill patients. The only modalities that entirely avoid risk to the patient are those continuous indices based on TCD or NIRS and ABP for which entirely non-invasive methodologies have been described (71–73).

The second key clinical consideration is the required spatial and temporal resolution required for the monitoring of CA. While imaging-based modalities provide great spatial resolution, they are limited to essentially capturing snapshots of cerebral autoregulatory function. This may be appropriate for the long-term outpatient monitoring of CA recovery following brain injury but does not provide the temporal resolution to potentially guide management in the acute care setting.

Contemporary utilization of CA monitoring in the clinical setting typically relies on continuous or semi-continuous modalities. These modalities provide limited spatial resolution but have a temporal resolution that is much more conducive to informing acute bedside care. They have become more commonly used, in recent years, in the setting of SAH and moderate and severe TBI as prognostic tools (74, 75). Beyond prognostication, these continuous modalities have begun to play a role in the management of critically ill patients. One such example of an invasive modality is the utilization of PRx to derive personalized CPP goals that maximize CA function in critically ill TBI patients. These targets are not only specific to the patient but are updated in real-time, thereby leveraging the high temporal resolution of continuous and semi-continuous modalities (76). Future work may improve the spatial resolution of the continuous modalities by measuring hemispheric CA using TCD or NIRS. Beyond this, the development of full scalp NIRS arrays, like those seen in fNIRS, may bring the spatial resolution of these continuous modulates inline with image-based intermittent methods (77).

A final consideration is the role CA monitoring plays in clinical research. The quantity and quality of data that can be extracted from each modality is important to understand when developing a clinical research project. As previously discussed, the radiographic and PET techniques only assess static autoregulation intermittently. This is ideal for studies that may want to elucidated regional differences in CA function at the steady state in both pathologic and healthy subjects. Given that they often required deliberate modulation of ABP they often have favorable signal-to-noise profiles reducing the number of trials required to identify a true signal. Modalities with high temporal resolution, such as some TCD-based techniques, enable the evaluation of dynamic CA. This is at the expense of spatial resolution as they are often only able to determine hemispheric differences in CA. These modalities can elucidate the rate at which CA acts in a dynamic fashion. Like radiographic/PET modalities, these measures often require induced perturbations in ABP and therefore have good signal-to-noise profiles. Lastly, semi-continuous and continuous modalities, often based on TCD or NIRS measures, allow for the evaluation of the evolution of CA function over long periods of time. As these methods rely on natural fluctuations in ABP they are noisy signal that require long periods of recording. These methods are ideal for studies that hope to examine the evolution of CA over time such as in the setting of aneurysmal SAH and moderate/severe TBI.

Ultimately, each modality has its own strengths and weaknesses. In order to select the most appropriate modality the clinician, or clinician-scientist, must have a thorough understanding of this. An informed selection of modality will improve patient care and enhance further research in this field.

## Author Contributions

AS and AG: undertook review and screening of the literature as well as aided in preparing the manuscript. LF, TS, CB, KS, DC, and AA: aided in preparation of the manuscript. FZ: responsible for conceptual development of this article, development of search strategy, undertook screening of the literature, and aided in preparation of the manuscript. All authors contributed to the article and approved the submitted version.

## Funding

This study was supported by the Manitoba Public Insurance (MPI) Neuroscience/TBI Research Endowment, the Health Sciences Centre Foundation Winnipeg, and the University of Manitoba Department of Surgery GFT Research Grant program. FZ receives research support from the Manitoba Public Insurance (MPI) Neuroscience/TBI Research Endowment, the Health Sciences Centre Foundation Winnipeg, the United States National Institutes of Health (NIH) through the National Institute of Neurological Disorders and Stroke (NINDS)(Grant #: R03NS114335-01), the Canada Foundation for Innovation (CFI)(Project #: 38583), Research Manitoba (Grant #: 3906), the University of Manitoba VPRI Research Investment Fund (RIF), the University of Manitoba Centre on Aging, and the University of Manitoba Rudy Falk Clinician-Scientist Professorship. AG was supported through the University of Manitoba Clinician Investigator Program, the University of Manitoba Dean's Fellowship, the Manitoba Medical Services Foundation Research and Education Fellowship, and the R. Samuel McLaughlin Research Fellowship. AS was supported through the University of Manitoba, Department of Surgery GFT Research Grant, and the UMGSA Scholarship at the University of Manitoba. LF was supported through the University of Manitoba-Department of Surgery GFT Research Grant, the University of Manitoba–University Research Grant Program (URGP), and the Biomedical Engineering Fellowship Grant at the University of Manitoba. TS was supported through the University of Manitoba-Department of Surgery GFT Research Grant.

## Conflict of Interest

DC is affiliated with the Pan Am Clinic Foundation which receives general education and research support from ConMed Linvatec, Ossur, Zimmer Biomet, and Arthrex. The remaining authors declare that the research was conducted in the absence of any commercial or financial relationships that could be construed as a potential conflict of interest.

## Publisher's Note

All claims expressed in this article are solely those of the authors and do not necessarily represent those of their affiliated organizations, or those of the publisher, the editors and the reviewers. Any product that may be evaluated in this article, or claim that may be made by its manufacturer, is not guaranteed or endorsed by the publisher.
